# Scleromyxedema: clinical diagnosis and autopsy findings*

**DOI:** 10.1590/abd1806-4841.20164527

**Published:** 2016

**Authors:** Ana Carolina Bulhões Sala, Paulo Rowilson Cunha, Clóvis Antônio Lopes Pinto, Célia Antônia Xavier de Moraes Alves, Ingrid Barreto Paiva, Ana Paula Vieira Araujo

**Affiliations:** 1 Universidade Estadual de Campinas (UNICAMP) – Campinas (SP), Brazil; 2 Faculdade de Medicina de Jundiaí (FMJ) – Jundiaí (SP), Brazil; 3 Private clinic – Vitória (ES), Brazil; 4 Private clinic – São Luís (MA) - Brazil

**Keywords:** Autopsy, Cyclophosphamide, Paraproteinemias, Scleromyxedema

## Abstract

Scleromyxedema is a rare chronic cutaneous mucinosis of unknown etiology. It is characterized by papular eruption and scleroderma with microscopic evidence of mucin deposition, fibroblast proliferation, and fibrosis. Most patients with scleromyxedema have monoclonal gammopathy and systemic manifestations resulting in significant morbidity and mortality. Several types of treatment have been reported with partial or inconsistent responses. Despite showing unpredictable evolution, systemic consequences of scleromyxedema and treatment side effects may result in death. We describe a rare case of a patient with scleromyxedema without paraproteinemia with systemic involvement that evolved to death despite treatment with cyclophosphamide.

## INTRODUCTION

Scleromyxedema is a rare chronic cutaneous mucinosis of unknown etiology. It affects adults between 30-70 years of age, with no gender preference. Typical clinical findings include: widespread papular eruption with sclerodermiform appearance; monoclonal gammopathy; absence of thyroid disease; and the histopathologic triad (dermal mucin deposition, fibroblast proliferation, and fibrosis). The absence of monoclonal gammopathy does not exclude the diagnosis of scleromyxedema, which is considered an atypical case.^1-3^

In addition to the skin changes, most scleromyxedema patients present with systemic manifestations that may involve cardiovascular, gastrointestinal, respiratory, skeletal, muscular, renal, and nervous systems, causing significant morbidity and mortality. The lack of high-quality studies on the effectiveness of treatments for scleromyxedema and the incomplete understanding of the pathogenesis of the disease have prevented the development of definitive guidance on the best treatment approach.^2,3^

## CASE REPORT

**Figure 1:**
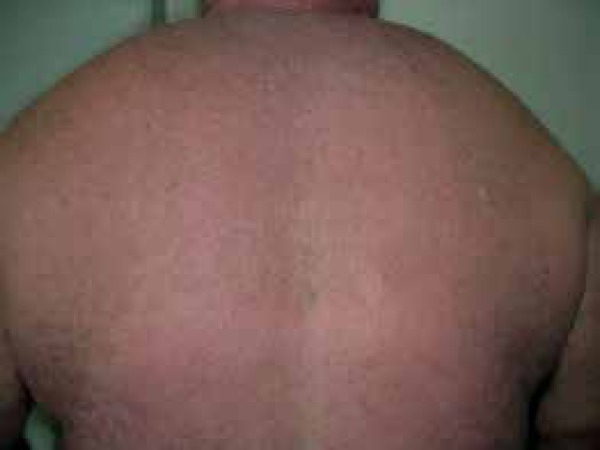
Papulareruption associated with erythema on the back and neck

**Figure 2:**
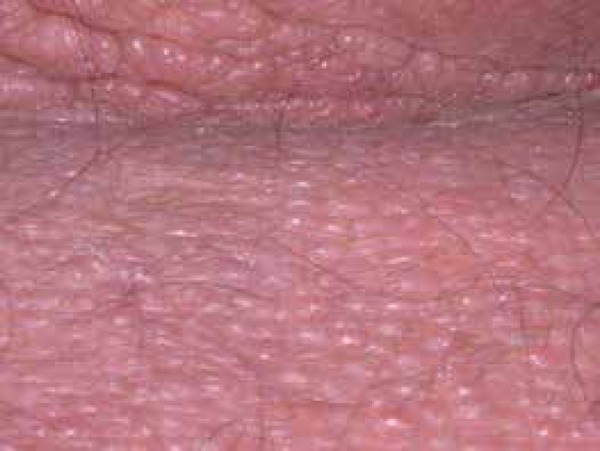
Coalescing normochromic papules, some in linear arrangement, and sclerodermiform surrounding skin

**Figure 3:**
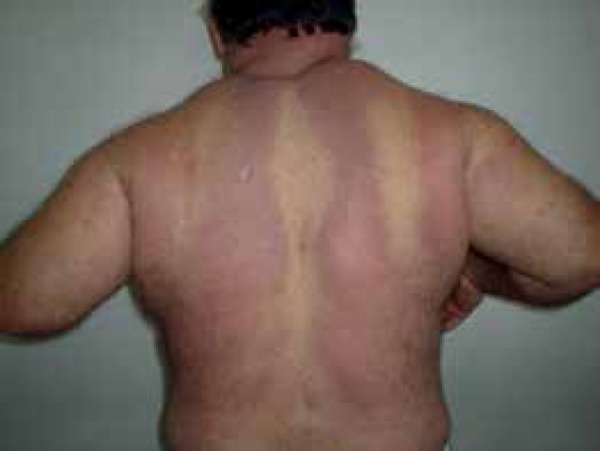
Erythematous areas interspersed with pallor and evidence of proximal weakness

A previously healthy 57-year-old male patient noted, five years before, hardening of the skin on the neck and back associated with local pruritus. He reports that one year after the symptoms appeared he developed dyspnea on mild exertion, dysphagia to solids, upper limb weakness, and polyarthralgia. Physical examination showed diffuse erythema associated with shiny coalescing normochromic papules involving the neck, upper trunk, and proximal portion of the upper limbs (Figure 1). Some papules showed linear arrangement with shiny and hardened surrounding skin (sclerodermiform) (Figure 2). When asked to raise his arms, he showed limited range of motion and pallor areas interspersed by erythema (Figure 3).

**Figure 4:**
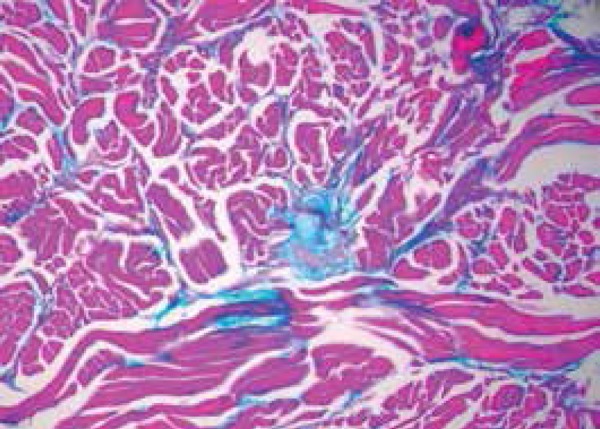
Positive colloi - dal iron staining, identifying mucopolysaccharides between the collagen bands (400x)

**Figure 5:**
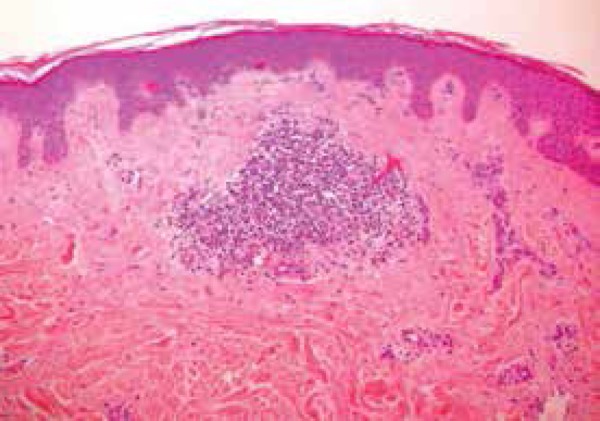
Dense superficial perivascular inflammatory infiltrates (HE, 100x)

A skin biopsy of a back sample revealed mucin deposition in the dermis between thick and dissociated collagen fiber bundles, suggesting the diagnosis of scleromyxedema. It also revealed dense superficial perivascular inflammatory infiltrates (Figures 4 and 5). Congo red and PAS stains were negative. Thyroid hormones were normal and protein electrophoresis with blood and urine immunofixation was negative for a monoclonal component. Echocardiography showed diastolic heart failure.

We chose to start treatment with cyclophosphamide. After six monthly sessions, however, the patient experienced clinical worsening and died. Autopsy revealed mucin deposition in the adventitia of the blood vessels of the heart, lungs, kidneys, and adrenal arteries. The cause of his death is unclear.

## DISCUSSION

Scleromyxedema is characterized by a symmetrical eruption of shiny firm papules measuring 2-3mm in diameter, which may be presented in a linear distribution. Papules may evolve to hardened plaques, showing marked sclerosis and hardening of the skin. It occurs predominantly on the face, neck, upper limbs, torso, and hands.^3,4^

In addition to the cutaneous findings, our patient presented with cardiac, gastrointestinal, and musculoskeletal systemic involvement. Musculoskeletal involvement is one of the most common extracutaneous manifestations; the patient reported arthralgia and muscle weakness, a common complaint in 90% of cases of scleromyxedema. Dysphagia, which was also reported by our patient, is the most common gastrointestinal manifestation and is linked to esophageal dysmotility arising mainly from the upper esophagus. Dysphagia is most commonly found in patients with associated myopathy, which was compatible with the present case. Cardiovascular abnormalities, such as congestive heart failure, may affect up to 10% of patients with scleromyxedema. Mucin deposition in the myocardium and coronary arteries has been previously reported.^3,5^

Although association with paraproteinemia occurs in over 80% of patients, its correlation to the severity or progression of the disease is not clear. Monoclonal gammopathy is usually IgG-lambda. However, scleromyxedema in the absence of paraproteinemia, as in the case described, is considered an atypical form of the disease.^3,5,6^

Histopathologically, scleromyxedema is characterized by mucinous deposits of a heterogeneous mixture of mucopolysaccharides acids that stain positive with alcian blue and colloidal iron, and are usually located in the mid and deep layers of the dermis. This deposit usually spares the papillary dermis and dissociates the collagen fibers.^7^

Currently, there is no consensus on optimal treatment of scleromyxedema because of the lack of randomized controlled trials, limited number of case reports, and incomplete understanding of the pathophysiology of the disease. Several methods have been used with varying success, including melphalan, interferon alpha, autologous stem cell transplant, thalidomide, cyclophosphamide, plasmapheresis, and intravenous immunoglobulin.^2,3^

Scleromyxedema is described as an unpredictable disease, but is usually progressive, debilitating, and lethal in the absence of a successful treatment. Death may result from complications with extracutaneous involvement or treatment side effects.^3^

Although our patient presented with a variety of cutaneous and systemic findings, the autopsy results were not correlated to the clinical picture. We observed no statistically significant mucinous deposits in the gastrointestinal tract, despite the symptoms; no mucinous deposits in renal and adrenals vessels were compatible with the clinical and laboratory findings. The lack of correlation between mucinous deposits and clinical features has already been reported. Montgomery described a patient presented with dyspnea and "mental depression" who had traces of mucin in the skin, which was not observed in any internal organs at autopsy.^8^ McCuiston, however, described a patient with weight loss, mental deterioration and psychosis. Autopsy revealed mucinous substance in the adventitia of the heart blood vessels and in the perivascular connective tissue of the kidney, adrenals, and pancreas.^9^ Despite the many neurological symptoms, we found no mucin deposition in the brain, only demyelination and gliosis. Therefore, different findings in patients with scleromyxedema indicate that mucin deposit can not be the cause of these events.^7,10^
